# Urological Melanoma: A Comprehensive Review of a Rare Subclass of Mucosal Melanoma with Emphasis on Differential Diagnosis and Therapeutic Approaches

**DOI:** 10.3390/cancers13174424

**Published:** 2021-09-02

**Authors:** Gerardo Cazzato, Anna Colagrande, Antonietta Cimmino, Concetta Caporusso, Pragnell Mary Victoria Candance, Senia Maria Rosaria Trabucco, Marcello Zingarelli, Alfonso Lorusso, Maricla Marrone, Alessandra Stellacci, Francesca Arezzo, Andrea Marzullo, Gabriella Serio, Angela Filoni, Domenico Bonamonte, Paolo Romita, Caterina Foti, Teresa Lettini, Vera Loizzi, Gennaro Cormio, Leonardo Resta, Roberta Rossi, Giuseppe Ingravallo

**Affiliations:** 1 Section of Pathology, Department of Emergency and Organ Transplantation (DETO), University of Bari “Aldo Moro”, 70124 Bari, Italy; anna.colagrande@gmail.com (A.C.); micasucci@inwind.it (A.C.); kcaporusso.c@libero.it (C.C.); mpragnellv@libero.it (P.M.V.C.); xeniatrabucco@hotmail.it (S.M.R.T.); andrea.marzullo@uniba.it (A.M.); gabriella.serio@uniba.it (G.S.); Teresa.lettini@uniba.it (T.L.); leonardo.resta@uniba.it (L.R.); roberta.rossi@policlinico.ba.it (R.R.); giuseppe.ingravallo@uniba.it (G.I.); 2 Section of Urology, Deparment of Emergency and Organ Transplantation (DETO), University of Bari “Aldo Moro”, 70124 Bari, Italy; marcellozingarelli86@gmai.com (M.Z.); alfonsolorusso@virgilio.it (A.L.); 3 Section of Legal Medicine, Interdisciplinary Department of Medicine, Bari Policlinico Hospital, University of Bari Aldo Moro, Piazza Giulio Cesare 11, 70124 Bari, Italy; mariclamarrone@hotmail.it (M.M.); alestellacci@gmail.com (A.S.); 4 Section of Ginecology and Obstetrics, Department of Biomedical Sciences and Human Oncology, University of Bari Aldo Moro, Piazza Giulio Cesare 11, 70124 Bari, Italy; francesca.arezzo@uniba.it (F.A.); Vera.loizzi@uniba.it (V.L.); Gennaro.cormio@uniba.it (G.C.); 5 Section of Dermatology, Department of Biomedical Sciences and Human Oncology, University of Bari Aldo Moro, Piazza Giulio Cesare 11, 70124 Bari, Italy; angela.filoni@gmail.com (A.F.); domenico.bonamonte@uniba.it (D.B.); Paolo.romita@uniba.it (P.R.); Caterina.foti@uniba.it (C.F.)

**Keywords:** melanoma, mucosal melanoma, urology

## Abstract

**Simple Summary:**

Mucosal melanoma accounts for only about 1% of all melanoma cases worldwide, but achieving the correct recognition, diagnosis and prognosis is of extreme importance for the patient. Moreover, primary melanoma of the urinary tract is a rarity among rarities, accounting for less than 4% of all the mucosal melanomas. The urethra is the most common location for this type of melanoma, followed by the bladder and ureter with decreasing incidences. Patients with this form of melanoma are usually elderly (over 60 years) and asymptomatic or have hematuria symptoms. Compared to other mucosal melanomas, there is not much knowledge of the risk factors, etiology, and molecular characteristics of urological melanoma. In addition, there are no uniform staging systems or treatment protocols available for this disease. A thorough knowledge of the existence and management of this disease entity is essential to allow research advances in this area and, in particular, to devise innovative therapies. In this paper, we focus in detail on melanoma originating from the uropoietic apparatus, discuss the cases reported in literature of this very rare and still partially enigmatic melanoma subclass, and consider the scenario leading to metastasization to neighboring organs.

**Abstract:**

Melanoma is reported as the 19th most common cancer worldwide, with estimated age-standardized incidence rates of 2.8–3.1 per 100,000. Although the origin is most frequently cutaneous, mucosal melanoma has been described several times in literature, and despite its rarity (only 1% of all melanomas), increasing attention is being paid to this disease form. Within this subgroup, melanomas of the uropoetic apparatus are a rarity among rarities. Indeed, less than 50 cases of primary melanoma originating from the urinary bladder have been described, and even less originating from the kidney, renal pelvis and urethra. In this work, we present a detailed review of the literature related to this subclass of mucosal melanoma, delve into the biological landscape of this neoplasm and discuss current approaches, future perspectives and potential therapeutic approaches.

## 1. Introduction

**Figure 1:**
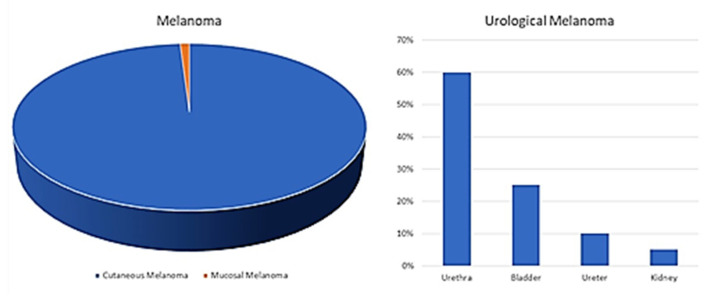
Primary melanoma of the urinary tract is a rarity among rarities (<4% of all the mucosal melanoma). The urethra is the most common location for this type of melanoma (50%), followed by the bladder and ureter with decreasing incidences [7].

Mucosal melanoma (MML) accounts for only 0.03% of all cancer diagnoses and 1.3% of all melanomas [1]. Typically, the median age at diagnosis is older: 67 years compared to 55 years for cutaneous melanoma [1]; about 65% of MML occurs in women. There does not seem to be a clear etiological association with ultraviolet (UV) rays, although no definitive results are yet available; trauma has been suggested as the possible etiological agent [2,3]. Among the different localizations, MML most frequently arises from mucous membranes of the head and neck (45–55%), anorectal region (25%), and genitourinary tract (15–20%) [4,5]. In both males and females, 5% of cases present in the distal urethra [6]. Primary urinary system melanomas are the least common among mucosal melanomas and account for less than 4% of all mucosal melanomas [1,6]. The urethra is the most common site for this type of melanoma, followed by the bladder and ureter (Figure 1). Patients with this form of melanoma are usually elderly (over 60) and asymptomatic or have hematuria symptoms [6,7]. The general approach for mucosal melanomas is wide resection surgery with lymph node dissection, followed by chemotherapy or immunotherapy [7]. However, the rarity of this neoplasm and difficulties in collecting large case series for observational and therapeutic studies have hampered the possibility of devising innovative therapies, including targeted therapies. Molecular studies of urinary tract melanoma are limited [7] although it has been shown that a small subgroup of primary urinary tract melanomas (about 14%) are characterized by the c-kit mutation, while the BRAF mutation is rare, being infrequently observed, unlike in cutaneous melanoma [7].

## 2. Materials and Methods

A systematic review was performed following the PRISMA guidelines (Preferred Reporting Items for Systematic Review and Meta-Analyses). Database searches were made of PubMed and Science.gov for the period 1952–2021 inserting the terms: melanoma, malignant melanoma in combination with each of the following: urology, kidney, ureter, bladder, urethra, penis, urinary system. Only articles written in English were selected. The last search date was 3 July 2021. Only articles including the histological diagnosis of urological melanoma were included. Patients with no definite histological diagnosis were excluded. The retrieved articles were assessed according to the 2011 Oxford Centre for Evidence-Based Medicine guidelines [8]. The selected articles included reviews, meta-analyses, observational studies, case reports, instant survey studies, letters to the editor and comments to letters. Other potentially relevant articles were identified by checking the references of the retrieved articles. The articles were selected independently by two researchers on the basis of the inclusion criteria. Any disagreement was resolved by discussion between the two researchers.

## 3. Results

In the literature search, a total of 149 records was initially identified, 19 of which were duplicates. After screening for the inclusion and admissibility criteria, 117 publications were finally included. The studies and their characteristics are shown in Table 1, Table 2, Table 3 and Table 4, ordered by topographic localization of the disease. Most of the selected publications were case reports (*n* = 88), followed by reviews (*n* = 18) and case series (*n* = 11). All the studies included were classified as level of evidence 4 or 5 for clinical research, as detailed in the 2011 Oxford Centre for Evidence-Based Medicine guidelines [8]. In total, 147 patients with a diagnosis of primary malignant melanoma (MM) of the urinary system were described.

Among these, 9 patients had primary/metastatic MM of the kidney and 7 patients primary/metastastic MM of the ureter, 43 patients had primary/metastatic MM of the bladder and 88 patients primary/metastatic MM of the urethra (males and females).

## 4. Discussion

Primary mucosal melanoma of the uropoietic apparatus is a very rare disease entity as compared to the cutaneous melanoma counterpart [1,2,3]; such forms account for just under 4% of all mucosal melanomas [7]. The sites most commonly affected are, in declining order, the kidney, ureter, bladder and urethra [7,9].

### 4.1. Kidney

Fujimoto et al. [9], Frasier et al. [10] and Tajima et al. [11] were the first authors to publish case reports of suspected primary MM of the kidney and/or renal pelvis in 3 patients. In 2002, Yildirim Bayazit et al. [12] described the third known case of primary MM of the kidney, originating from the medial lobe, in a 37-year-old patient. This patient had previously undergone fine needle aspiration cytology (FNAC), which had revealed a cell population of uncertain classification. The patient underwent therapy with Interferon 2b but died of systemic progression of the disease.

Later, in 2011, a new report was made by Tasdemir et al. [13] of a case of MM of the kidney, incidentally discovered in a 67-year-old male. The question as to whether primary MM of the kidney really exists had by then been much debated among dermatopathologists and uropathologists. It was discussed in 2000, in a paper by Ribalta et al. [106], who reported the clinical case of a 73-year-old man who presented with a hemorrhagic renal tumor initially interpreted as renal cell carcinoma (RCC). Then, after he suffered a retroperitoneal recurrence infiltrating the duodenal wall, immunohistochemistry showed positivity to HMB45, S-100 protein, actin and vimentin. The authors proposed considering this kidney cancer as a malignant epithelioid, pigmented, clear cell (“sugar”) variant of angiomyolipoma, rather than a primary MM of the kidney, raising doubts about the existence of this latter. In this context, a complete medical history and accurate dermatological, ophthalmological and internal medicine studies are fundamental to exclude any possibility that an unidentified, regressed melanocytic lesion could actually be responsible for the kidney involvement. Criteria have been developed, originally for MM of the bladder, but that are well suited also to all types of mucosal melanoma. Ainsworth et al. [107] and Stein-Kendall [108] stressed the importance of ascertaining any previous or current history of cutaneous melanoma and making a meticulous analysis of the entire cutaneous surface (including the use of a Woods lamp to exclude the presence of any depigmented area indicating regression of a possible melanoma). Necessary examinations include a thorough clinical study to exclude any ophthalmic or visceral site of onset, and microscopic study of any atypical melanocytes present in the mucosa adjacent to the main tumoral mass. When one or more of these criteria are present, then a metastatic melanoma may be hypothesized. The authors Cunningham et al. [15], Boughan et al. [16], Klatte et al. [17] and Levin et al. [18] reported cases of metastatic MM of the kidney of an ascertained primary nature. This is the essential watershed in this field. In the paper by Levin et al. [18], the very first case of renal metastasis of a primary ocular melanoma was reported, 20 years after the histological diagnosis. In conclusion, it seems to be absolutely essential to fully reconstruct the patient’s history, and to remember that one of the greatest risks of misdiagnosis for the histopathologist is in cases of “epithelioid” and/or atypical variants of renal angiomyolipoma, which can be taken for MM. This risk is well known and described in the literature [109,110]. Table 1 summarizes all studies related to kidney melanoma.

### 4.2. Ureter

More than 90% of cases of melanoma of the ureter are metastatic localizations of a previous cutaneous melanoma [6,7], although case histories and anecdotes have been reported, describing primary lesions likely originating from the ureteral mucosa.

One of the very first cases in the literature dates back to 1962, when Judd et al. [19] described the case of a 63-year-old man with no history of cutaneous melanoma, who developed a primary lesion of the ureter. Only after autopsy was this revealed to be secondary to a previous lesion of the superior renal calyx in the right kidney. After another case described by Garcia et al. in 1969 [20], more recently (2016), Khan et al. [21] described the case of a 78-year-old man with hematuria. Following cystectomy, histology revealed a synchronous melanoma of the bladder and ureter. In this paper, the authors highlighted the importance of multidisciplinary therapeutic management for this type of lesions, although therapy has not yet been standardized. Gakis et al. [22], Macneil et al. [23] and March et al. [24] described cases of metastatic involvement of the ureters in cases of primary cutaneous MM: this is the scenario most commonly observed. Table 2 summarizes all studies related to ureter melanoma.

### 4.3. Urinary Bladder

**Figure 2:**
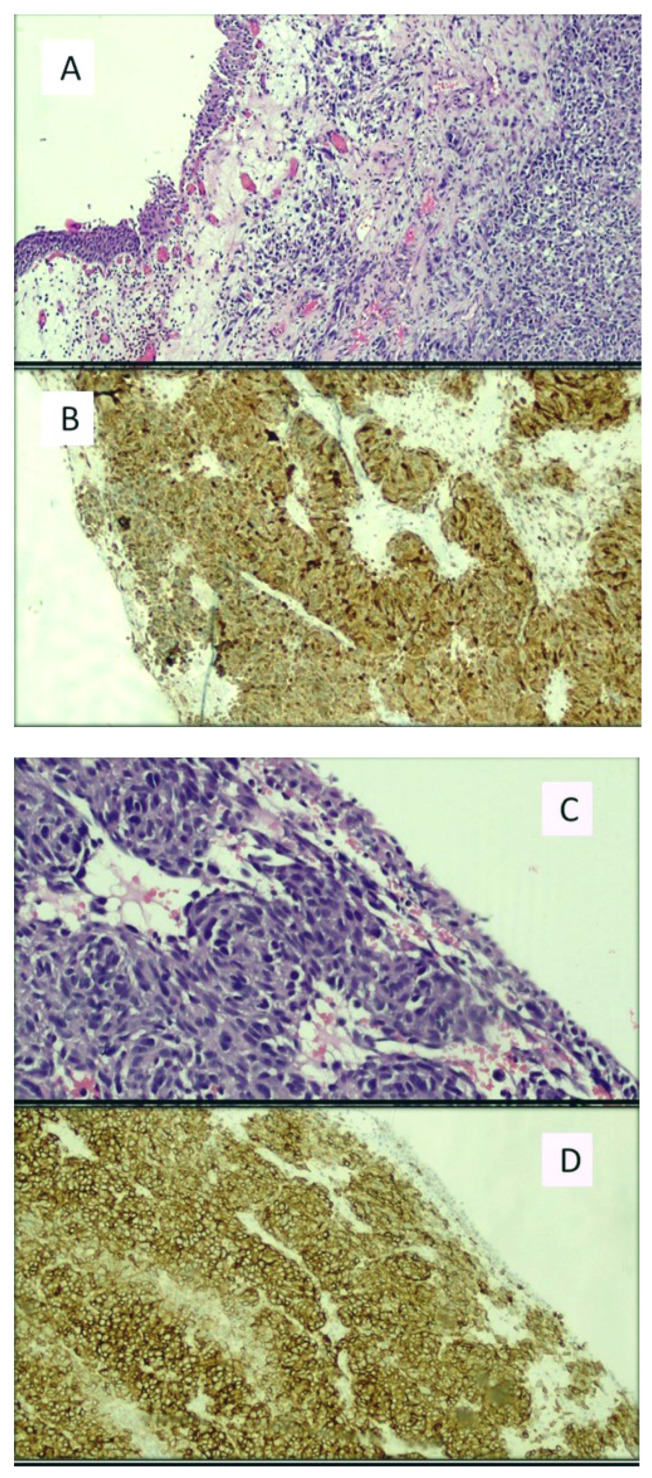
(**A**) Histological micrograph including urothelial mucosa and subepithelial proliferation of pleomorphic cells disposition, with atypia and nuclear pleomorphism but without evident presence of melanic pigment. (Hematoxylin-Eosin, Original Magnification: 10×). (**B**) The proliferation was shown to be constituted by melanocytes immunoreactive to the anti-HMB-45 antibody (Immunohistochemistry, Original Magnification: 10×). (**C**) Histological detail of the bladder mucosa including melanocytes proliferation nested around the blood vessels. (Hematoxylin-Eosin, Original Magnification: 20×). (**D**) Diffuse, strongly positive immunoreaction to the anti-HMB45 antibody. (Immunohistochemistry, Original Magnification: 20×). The sample belonged to a patient with primary bladder melanoma who died a few months after the histological diagnosis. Molecular investigations revealed the presence of the BRAFV600E mutation.

The earliest cases of primary melanoma of the bladder in literature were published in 1980, when Willis et al. [25] and Anichkov and Nikonov (1982) [26] reported the first four patients affected by a presumed bladder MM, with no clinical history of primary cutaneous melanocytic neoplasia. Then, in 1988, adding to the very few cases yet described, Goldschmidt et al. [27] detailed their experience of a 53-year-old woman who complained of persistent hematuria that had lasted some time, although she did not remember precisely how long. Urography and cystoscopy revealed a “mushroom-like” lesion on the bladder wall, and after targeted biopsy, the diagnosis of MM was made and radical cystectomy was performed. After 6 months of close follow-up, no recurrence had been observed. In the second case reported by the same authors, a 56-year-old woman presented with a nodule at the angle of the mandible. Biopsy demonstrated a proliferation of melanocytic cells attributable to melanoma. Following histomorphological and ultrastructural studies, the authors concluded that it was a subcutaneous metastasis of a primary melanoma originating in the bladder mucosa. An example of bladder melanoma (found during our practice) is presented in Figure 2A–D.

In 1992, Van Ahlen H. et al. [28] reported the clinical case of an 82-year-old patient who had been referring to their outpatients clinic for some years for bladder symptoms. Initially, these had been misinterpreted as due to an *Escherichia coli* infection, but antibiotic therapy had not resolved nor improved the symptoms in any way. Cystoscopy demonstrated a papillary lesion at the base of the bladder, with no anomalous pigmentation at macroscopic examination. The patient underwent transurethral resection of the bladder trigon (TURBT). Histology revealed a solid tumor with many necrotic areas, showing positivity to S-100 protein and MM was therefore diagnosed. Another case was reported in 2011 by Ammari El. et al. [31], and then in 2012, Schindler et al. [32] described the only case (until now) of primary melanoma of the urinary bladder, with a rhabdoid phenotype, in a female patient. She was treated with the anti-CTLA4 antibody Ipilimumab, which yielded a partial response. In the same paper, the diagnostic problems of this very rare disease were discussed, and the possibility was highlighted that at immunohistochemistry (IHC) the tumoral cells could be positive for S-100 protein, Vimentin and CD56 (NCAM), and negative for routine melanocytic markers such as Melan-A and HMB-45, was highlighted. Other reports of primary melanoma of the bladder were made in the following years [29,30,33,34,35,36,37,38]. Very recently, in 2021, Snajdar E. et al. [39] reported the case of a 78-year-old woman who presented to the Emergency Department for a new onset of incontinence and macroscopic hematuria. After the first examination and various tests, she underwent TURBT. Histopathologic analysis of the tumor revealed a primary MM of the bladder, in which the tumor cells stained positive for HMB-45 and S-100 protein. After the histopathologic result, fluorodeoxyyglucose (FDG) positron emission tomography (PET) was performed, yielding positive results only in the bladder and negative imaging of the chest. The diagnosis of a primary MM of the bladder was confirmed. After about 8 months, the patient underwent radical cystectomy and pelvic lymph nodes dissection; histology confirmed the previous TURBT diagnosis. A few months later, the patient died of metastatic disease progression. Various reports [40,41,42,43,44,45,46,47,48,49,50] have dealt with cases of secondary involvement of the bladder by a previous cutaneous skin melanoma. In one of the most recent of these papers, Topal et al. [48] reported their experience of a 70-year-old patient who had presented to the Urology Department for macrohematuria. Both the TURBT, and the later operative specimen from radical cystectomy, were positive for MM, that appeared to be primary. However, close study and reconstruction of the clinical history demonstrated that 15 years before, the woman had undergone enucleation of the left ocular bulb for primary ocular MM. This case was, therefore, the third report in literature of this very rare occurrence [45,46]. In light of the results of this review of MM of the urinary bladder, we can state that we believe the above-mentioned criteria for the kidney, proposed by Ainsworth et al., remain valid today. These authors consider that primary MM of the bladder is a diagnosis based on exclusion, to be made only after having very carefully reconstructed the entire personal and family history of the affected patient.

It is important, in our view, to underline the lack of agreement in the scientific community also as regards whether primary melanoma of the bladder actually exists. For example, in 2000, García Montes et al. [111] presented the case of a 30-year-old patient with a diagnosis of suspected primary melanoma of the bladder, discussed the relatively stringent criteria for such a diagnosis, but concluded that no test could definitively demonstrate that there had been no previous complete regression of a hypothetical primary cutaneous melanocytic lesion that could no longer be identified.

As regards the best treatment, it should be remembered that the prognosis of primary and/or metastatic melanoma of the bladder is always poor. Some more recent works have attempted to define a therapeutic algorithm [112]. In 2019, Chaus et al. [52] reported the clinical case of a 27-year-old woman with a family history of melanoma and evidence of a malignant melanoma of the urinary bladder. After TURBT, various instrumental examinations such as CT/abdominal-pelvic MRI and total-body PET were negative for distant recurrence, so the CARIS molecular intelligence tumor profiling was characterized. This showed positivity for the following mutations: BRAF V600E, PTEN Exon 9, PDL1-2+, and a high tumor mutation burden. The patient was therefore subjected to partial robot-assisted cystectomy and immunotherapy with Pembrolizumab, a PD-1 anti-immune checkpoint. During the subsequent 2-year follow-up, the patient was disease-free and showed no signs of major toxicity. Recently, in 2021, Rapisarda et al. [55] described the clinical history of a 74-year-old man with primary melanoma of the bladder. He was treated with TURBT plus intravesical chemotherapy with Bacillus Calmette-Guérin (BCG), that offers a further treatment option for this neoplasia, but only in selected cases.

To conclude, it must be borne in mind both that the survival rate at three years in patients affected by bladder melanoma is very low, and that the disease is so rare that a standardized treatment protocol is still lacking. Table 3 summarizes all studies related to bladder melanoma.

### 4.4. Urethra and Penis

One of the first reports in literature of a primary melanoma of the urethra or penis in a male patient dates back to 1976, when Konigsberg H. et al. and Gray G.F. et al. [56] presented two rare cases, one of penile melanosis and the other of a primary melanoma of the penis. The authors underlined the rarity of both clinical pictures, the clinical difficulties of making a differential diagnosis between the two conditions (the first of which is benign but the second overtly malignant) and suggested what might be the best therapeutic approaches (at the time). After that, growing numbers of reports were made [59,60,61,62,63,64,65,66,67]. Then, in 1993, Rashid et al. [66] reported two cases of primary melanoma, one of the male urethra and one of the penis, in two middle-aged subjects. They probed the difficulties in diagnosing such lesions. Although some suggestions had already been made (such as staining for melanin according to Fontana-Masson), the authors stressed the different biological behavior of melanocytic lesions at the genital level and discussed the various treatment options available at the time. The survival rate of patients affected by primary melanoma of the urethra or penis was very low (<5% at 3 years) [69], although in 1996 a patient with urethral melanoma was reported, who unexpectedly survived, disease-free, for several years [70].

In 2005, Ortis S. et al. [76] reported their experience of 10 patients, enrolled over a period of about 42 years (1962–2000), affected by primary melanoma of the penis/urethra, as well as 6 patients affected by primary melanoma of the scrotal skin. They evaluated the clinical and pathologic characteristics, the Breslow thickness, surgical treatment and clinical course. Of the 10 patients enrolled, 4 had stage T1 (according to the American Joint Committee on Cancer), with a depth of less than 0.75 mm; 3 had stage T2 (0.75–1.5 mm) and 3, stage T3 (1.51–4 mm). In only one of 4 cases with palpable inguinal lymph nodes did lymphadenectomy (BILND) demonstrate inguinal lymph node metastases. In 7 patients with stage T1-2N0M0, no local recurrence occurred after wide local excision (WLE) or partial penectomy, after a mean follow-up of 35 months. Six of the seven men remained disease-free. In all the patients with melanoma of the penis, the specific actuarial survival and disease-free rates at 5 years were 80% and 60%, respectively, at a median follow-up of 39 months (range 20 to 210). The six patients with scrotal melanoma were treated with WLE and no local recurrence occurred. Three of the 6 patients presented palpable inguinal lymph nodes, and 2 of them died after chemotherapy for non resectable disease, while 1 died of other causes, 51 months after a negative BILND. The 3 men with clinically negative inguinal lymph nodes, who did not undergo BILND prophylaxis, developed distant (1) or regional metastases (2) and died of disease progression. In the patients with scrotal melanoma, the specific actuarial survival and disease-free rates at 5 years were 33.3% and 33.3%, respectively, after a median follow-up of 36 months.

Many other reports [77,78,79,80,81,82,83,84,85,86,87,88,89,90,91,92,93,94,95,96,97,98,113,114,115,116] have described cases of primary melanoma of the penis/male urethra. Instead, in 2020, Naktra et al. [100] presented a curious case of primary melanoma of the female urethra with neuroendocrine differentiation, in a 62-year-old woman with urinary obstruction symptoms. Clinical and radiological examination revealed a large urethral mass with liver and lymph nodes metastases. Biopsies were made of the urethral and hepatic lesions, demonstrating poorly differentiated tumor cells with a small cell morphology and the presence of melanic pigment. These cells were immunopositive for melanocytic and neuroendocrine markers. Ultrastructural examination demonstrated the presence of melanosomes and neurosecretory granules in the tumor cells. This was the very first case report of this disease entity originating in the female urethra (in itself extremely rare, accounting for 0.2% of all forms of melanoma) with a “small cell” appearance at histology.

Still more recently (2021), Burity et al. [105] presented a case of primary melanoma of the urethra in a 79-year-old male patient, with a blackened lesion of the urethral meatus, 1.5 cm in size. Biopsy demonstrated a malignant neoplasia, and the patient underwent partial penectomy, with 2 cm excision margins, and deep bilateral lymphadenectomy. No disease recurrence was observed at follow-up. In the conclusions, the authors underlined the fact that the tumoral mutation burden of melanoma in uncommon sites such as the urethra/penis is lower overall than in sunrays-exposure cutaneous melanoma. This can also influence the response rate, to immunotherapy, for example. Moreover, in mucosal melanoma a distinct model of chromosomal aberrations has been reported, as well as a higher rate of copy number alterations, and frequent KIT mutations in urogenital melanomas. Table 4 summarizes all studies related to urethra and penis melanoma.

Finally, in 2020, a series of authors developed UK national guidelines [117], aiming to make the diagnostic-therapeutic assistance (PDTA) pathways of patients with uro-genital tract melanoma more uniform, clear and easily accessible, starting from the evidence in the literature. After briefly summarizing the evidence from case reports and case series present in the literature and discussing the main histomorphological and immunophenotypic characteristics, as well as molecular biology, the authors underline that the best treatment approach aimed at modifying the (rather low) survival rates of patients with MM should be based on the most up-to-date guidelines, and in this regard their recommendations are to: (1) use single-agent programmed cell death protein 1 (PD1) antibodies in patients with unresectable stage III or stage IV tumors, but taking into account any contraindications to this therapy; (2) consider combination immunotherapy, for example, anti-CTLA (cytotoxic T-lymphocyte-associated protein and anti-PD1/PD-L1 (programmed cell death ligand monoclonal antibodies in selected, fit patients; (3) consider BRAF + MEK inhibitors as a treatment option for the small number of patients with BRAF mutated unresectable stage III or stage IV MM: (4) in all cases where there is no clearly predominant risk/benefit ratio for any therapeutic approach, inform the patient about all the existing possibilities and choose together.

## 5. Conclusions

Due to the rarity of these disease entities, their diagnosis and treatment still pose a challenge. Primary melanoma of the urological tract is a very rare and aggressive neoplasm. Despite the controversies that have arisen about the appropriate surgical treatment, early diagnosis and treatment are essential to allow a successful clinical outcome and patient survival. Targeted therapy may be a good treatment alternative for this rare and poorly diagnosed tumor type. For example, in one of the most recent works in the literature [7], it is clearly demonstrated how next generation sequencing is opening up new avenues for studying mutations also in this subgroup of pathology. In particular, mutations were found in more recurrent genes as also BRAF V600E, BRAF V600K, but also amplifications of genes such as ERBB2, FGFR1, and MET. In addition, a subset of patients had KIT gene mutations. Considering the potential difficulty in diagnosis and the lack of an optimized management algorithm, we believe that more research, including the identification of molecular targets, will add value to the literature on the diagnosis and treatment of urinary melanoma.

## Figures and Tables

**Table 1:** Papers in literature related to primary/metastatic melanoma of the kidney.

Author(s)	Year(s)	Primary/Metastatic	Treatment	Survival
Fujimoto, H. et al. [9]	1995	Primary	PRK and CHT	44 months
Frasier, B.L. et al. [10]	1988	Primary, renal pelvis	TRK and AI	22 months
Tajima, K. [11]	1997	Primary	TRK	2 years and 3 months
Hor Bayazit, Y. et al. [12]	2002	Primary	TRK and LY	1 year
Tasdemir, C. et al. [13]	2011	?	TRK	some months
Agnew, C.H. et al. [14]	1958	Metastatic	TRK	some months
Cunningham, J.A. et al. [15]	1994	Metastatic	CHT, HT and IT	6 weeks
Boughan, K.M. et al. [16]	2009	Metastatic	Not reported	10 days
Klatte, T. et al. [17]	2007	Metastatic, like renal vein thrombus	TRK	5 months
Levin, B.M. et al. [18]	2005	Metastatic, from ocular melanoma	TRK	still alive

Legend.: PRK: partial resection of kidney; TRK: total resection of kidney; LY: lymphadenectomy; CHT: chemotherapy; HT: P-CHT: postoperative chemotherapy; I: immunotherapy; AI: adjuvant immunotherapy.

**Table 2:** Papers in literature related to primary/metastatic melanoma of the ureter.

Author(s)	Year(s)	Primary/Metastatic	Treatment	Survival
Nguyen, A.T. et al. [6]	2001	Metastatic (13 patients)	CHT, IT and/or HT	median survival (43 months)
Acikalin, A. et al. [7]	2020	Metastatic (8 patients)	CHT, IT, HT and/or TT	median survival (<3 years)
Judd, R.L. [19]	1962	Primary	SH	5 months
Garcia, A.E. et al. [20]	1969	Primary	CHT	<6 months
Khan, M. et al. [21]	2016	Primary	/	death before starting therapy
Gakis, G. et al. [22]	2009	Primary	CHT and SH	3 months
Macneil, J. [23]	2016	Primary	ER	after 12 months still alive
March, B. et al. [24]	2017	Metastatic, obstruction	/	/

Legend.: CHT: chemotherapy; IT: immunotherapy; HT: hormonal therapy; TT: target therapy; ER: endoscopic resection; SH: surgery.

**Table 3:** Papers in literature related to primary/metastatic melanoma of the bladder excluding reviews without case presentation.

Author(s)	Year(s)	Primary/Metastatic	Treatment	Survival
Willis, A.J. et al. [25]	1980	Primary	CHU and CHT	<6 months
Anichkov, N.M. et al. [26]	1982	Primary (2 patients)	both CH and CHT	1 year, lost to FU
Goldschmidt, P. et al. [27]	1988	Primary (2 patients)	both CH and CHT	(1) still alive after 6 months, (2) 7 months
Van Ahlen, H. et al. [28]	1992	Primary	CH, IT and CHT	still alive after 15 months
Lange-Welker, U. et al. [29]	1993	Primary	CHU and CHT	some months
Pacella, M. et al. [30]	2006	Primary	PCH and CHT	9 months
El Ammari, J.E. et al. [31]	2011	Primary	CH	<6 months
Schindler, K. et al. [32]	2012	Primary, with rhabdoid features	CH, CHT and IT (anti CTLA4)	12 months still alive
Truong, H. et al. [33]	2013	Primary, recurrent	CHU and IT (anti CTLA4)	still alive
Karabulut, Y.Y. et al. [34]	2016	Primary, 5 cases	CH and 2 patients IT	2 died after some months, 3 stil alive
Laudisio, A. et al. [35]	2016	Primary, older man	Only CHT	after 12 months still alive
Buscarini, M. et al. [36]	2017	Primary	CH and IT	some months
Singh, V. et al. [37]	2019	Primary, with monster cells	CH and IT with Nivolumab	still alive
Kirigin, M. et al. [38]	2019	Primary	TURBT	death after few weeks
Snajdar, E. et al. [39]	2021	Primary	CH and adjuvant CHT	16 months
Maeda, T. et al. [40]	2008	Metastatic	CH, CHT and IT	some months
Nohara, T. et al. [41]	2009	Metastatic	TURBT and CHT	some months
Siroy, A.E. et al. [42]	2011	Primary	TURBT and CH	some months
Efesoy, O. et al. [43]	2011	Metastatic	TURBT and CHT	7 months
Meunier, R. et al. [44]	2015	Metastatic	TURBT, CH and CHT	some months
Theocharides, C. et al. [45]	2017	Metastatic	TURBT and IT	some months
Paterson, A. et al. [46]	2012	Metastatic	CH and CHT	<5 months
Ikeda, A. et al. [47]	2013	Metastatic	TURBT and CHT	<5 months
Topal, C.S. et al. [48]	2016	Metastatic	CH and CHT	some months
Patil, R.V. et al. [49]	2017	Metastatic	CHU, SRT and IT with anti CTLA4	4 months
Barillaro, F. et al. [50]	2018	Metastatic	CH and IT with Nivolumab	still alive
Nair, B.C. et al. [51]	2011	Metastatic, from conjunctival melanoma	CH, CHT and IT	some months
Chaus, F.M. et al. [52]	2019	Metastatic	RPCH and IT with Pembrolizumab	still alive after 2 years
Mercimek, M.N. et al. [53]	2019	Metastatic	TURBT, LPC and IT	still alive
Moez, R. et al. [54]	2020	Metastatic	TURBT, CH and CHT	still alive
Rapisarda, S. et al. [55]	2021	Metastatic	TURBT, CH and BI	still alive after 6 months

Legend: TURBT: trans-urethral resection bladder tumor; CH: cystectomy; PCH: partial cystectomy; CHU: cystourethrectomy; RPCH: robotic partial cystectomy; LPC: laparoscopic partial cystectomy; CHT: chemotherapy; IT: immunotherapy; SRT: stereotactic radiotherapy (for brain metastases); BI: bladder instillations; FU: follow-up.

**Table 4:** Papers in literature related to primary/metastatic melanoma of the urethra and/or penis, excluding reviews without case presentation.

Author(s)	Year(s)	Primary/Metastatic	Gender	Treatment	Survival
Konigsberg, H.A. et al. [56]	1976	Primary, urethra and penis	2 M	UTH and PH UTH and PH	20 years <6 months
Kokotas, N.S. et al. [57]	1981	Primary, urethra	M	PH	6 months
Barbagli, G. et al. [58]	1988	Primary, urethra	F	UTH and CH	1 year
Yamamoto, N. et al. [59]	1989	Primary, urethra	M	PCH	6 months
Primus, G. et al. [60]	1990	Primary, urethra and penis	M	UTH and PH	>6 months
Calcagno, L. et al. [61]	1990	Primary, urethra	M	UTH and CHT	>6 months
Fujimoto, N. et al. [62]	1991	Primary, urethra	M	UTH and CHT	4 months
Ander, H. et al. [63]	1991	Primary, urethra	M	UTH and RT	>6 months
Arai, K. et al. [64]	1993	Primary, urethra	F	UTH and RT	1 year
Kim, C.J. et al. [65]	1993	Primary, urethra	F	UTH and CHT	5 years
Rashid, A.M. et al. [66]	1993	Primary, urethra and penis	2 M	UTH UTH and CHT	<6 months <6 months
Aragona, F. et al. [67]	1995	Primary, urethra	F	UTH and CHT	6 months
Gincherman, Y. et al. [68]	1996	Primary, distal urethra	M	UTH and CHT	5 years
Touyama, H. et al. [69]	1997	Primary, urethra	F	UTH and CHT	After 4 months still alive
Girgin, C. et al. [70]	1999	Primary, urethra	F	UTH	<6 months
Watanabe, J. et al. [71]	2000	Primary, urethra	M	UTH and CHT	>6 months
Chitale, S.V. et al. [72]	2001	Primary, urethra	M	PUTH	>6 months
Kubo, H. et al. [73]	2002	Primary, urethra	M	PUTH and IT	>6 months
Mukai, M. et al. [74]	2003	Primary, urethra	F	PUTH, UTH and IT	>6 months
DiMarco, D.S. [75]	2004	Primary, urethra	11 F/M	PUTH, UTH, CHT and IT	Median survival: 7 months
Sánchez-Ortiz, R. et al. [76]	2005	10 patients: primary of urethra and/or penis 6 patients: primary of scrotum	M	PUTH and/or UTH and/or CHT and/or IT	Median survival: 9 months
Katz, E.E. et al. [77]	2005	Primary, urethra	M	UTH and CHT	6 months
Kato, H. et al. [78]	2005	Primary, urethra	M	UTH and CHT	>6 months
Yoshizawa, T. et al. [79]	2007	Primary, urethra	F	UTH, CH and CHT	14 months
Nakamoto, T. et al. [80]	2007	Primary, urethra	F	UTH, CH and HY	>6 months
Inoue, M. et al. [81]	2008	Primary, urethra	M	PH and PRH	7 months
Comploj, E. et al. [82]	2009	Primary, urethra	M	UTH and IT	after 5 years still alive
Akbas, A. et al. [83]	2010	Primary, urethra	F	UTH and CH	13 months
Yoshii, T. et al. [84]	2010	Primary, urethra	F	UTH, CH and CHT	25 months
Cho, S.T. et al. [85]	2012	Primary, urethra	F	UTH	After 6 months still alive
Karaman, H. et al. [86]	2013	Primary, urethra	M	UTH	>6 months
Maruyama, T. et al. [87]	2014	Primary, urethra	F	UTH and CH	11 months
Papeš, D. et al. [88]	2014	Primary, urethra and penis	Review	UTH and/or CH and/or PCH and/or CHT and/or IT	Median survival: 28 months
Li, Y. et al. [89]	2014	Primary, urethra and penis	M	UTH, PH, CHT and IT	After 3 years still alive
Pandey, P.K. et al. [90]	2014	Primary, urethra	F	UTH and CHT	>6 months
Broussard, A.P. [91] and McComiskey, M. [88]	2015 2015	Primary, urethra Primary, urethra	F F	UTH and CHT UTH and CHT	>6 months >6 months
Safadi, A. et al. [92]	2017	Primary, urethra	F	UTH and CH	/
Suzuki, H. et al. [93]	2018	Primary, urethra	F	UTH, CH and CHT	19 months
Davuluri, M. et al. [94]	2019	Primary, urethra	M	UTH, CH and CHT	11 months
Aoki, Y. et al. [95]	2019	Primary, urethra	M	UTH and PH	After 6 months still alive
Tokita, T. et al. [96]	2018	Primary, urethra	M	UTH, PH and IT with Nivolumab	After 20 months still alive
Maruyama, Y. et al. [97]	2018	Penile foreskin	M	UTH and PH	After 2 years still alive
Bansal, N. et al. [98]	2018	Primary, urethra	F	UTH	After 3 months still alive
Hansen, M.F. et al. [99]	2019	Primary, urethra	F	UTH	1 years and 8 months
Nakra, T. et al. [100]	2020	Primary, urethra with small cells	M	UTH and IT	>6 months
Sun, X. et al. [101]	2020	Primary, urethra	F	UTH	>6 months
Watanabe, K. et al. [102]	2020	Primary, urethra	F	UTH, HY and CH CHT and IT with nivolumab and ipilimumab	22 months
Kaboré, F.A. et al. [103]	2020	Primary, urethra	F	UTH and CH	After 1 year still alive
Zeighami, S. et al. [104]	2020	Primary, urethra	M	UTH and CH	>6 months
Burity et al. [105]	2021	Primary, urethra	M	UTH and PH	After 6 months still alive

Legend: UTH: urethrectomy; PUTH: partial urethrectomy; PH: penectomy; CH: cystectomy; PCH: partial cystectomy; HY: hysterectomy; PRH: prostatectomy; CHT: chemotherapy; IT: immunotherapy; RT: radiotherapy.

## Data Availability

Not applicable.
